# Alcohol Withdrawal

**DOI:** 10.21980/J87S8Q

**Published:** 2025-01-31

**Authors:** Patrick Meloy, Dan Rutz, Amit Bhambri

**Affiliations:** * Emory University School of Medicine, Department of Emergency Medicine, Atlanta, GA; ^ Utah Emergency Physicians, P.C. Murray, Utah; † Swedish Covenant Hospital, Department of Emergency Medicine, Chicago, IL

## Abstract

**Audience:**

Emergency medicine residents and medical students on emergency medicine rotations

**Introduction:**

Alcohol use disorder (AUD) is common in the United States, with an estimated lifetime prevalence of 30%.^1^ The rate of use is higher among white males, Native Americans, and individuals of low socioeconomic status.^1^ Alcohol withdrawal symptoms manifest in 50% of individuals who misuse alcohol.^1^ While life-threatening sequelae of alcohol withdrawal are rare, the syndrome is a common reason for emergency department (ED) presentations. Alcohol withdrawal symptoms range from benign, cravings, nausea, anxiety and tremulousness, to life-threatening autonomic dysfunction, seizures, coma, and death.^2^ The pathophysiology of this clinical syndrome involves dysregulation of central nervous system (CNS) receptor function. Alcohol acts as a CNS depressant through activation of the CNS Gamma-aminobutyric acid (GABA) receptors. Chronic or heavy alcohol use results in downregulation of CNS inhibitory GABA receptors and upregulation of CNS excitatory *N*-methyl-D-aspartate (NMDA) receptors.^2^ Upon discontinuation of alcohol use, this imbalance results in CNS hyperexcitability, creating the clinical symptoms of alcohol withdrawal.^2^ Symptoms typically manifest within eight hours after alcohol cessation, reach their peak in one to three days, and can extend for up to two weeks.^3^ Mild symptoms include anxiety, tremors, diaphoresis, nausea and/or vomiting. Severe symptoms include hallucinations (typically 12–24 hours after last alcohol intake) in 2–8% of patients, seizures (12–48 hours after last intake) in up to 15% of patients, and delirium tremens.^3^ Delirium tremens is a potentially fatal encephalopathy in patients experiencing alcohol withdrawal and occurs in 3–5% of patients approximately 72 hours after last alcohol intake.^3^ Without recognition or prompt treatment, mortality from delirium tremens can be as high as 50%.^4^ Management of alcohol withdrawal requires prompt recognition and control of symptoms. Most often this is accomplished by administering benzodiazepines, though alternative medications such as barbiturates, ketamine, or propofol are also used. Severe withdrawal may progress to intubation and mechanical ventilation.^5^ Given the high prevalence of AUD in the United States and the potential for life-threatening withdrawal symptoms, ED practitioners must recognize the spectrum of this disease and be comfortable with managing an array of presentations.

**Educational Objectives:**

At the end of this oral boards session, learners will: 1) demonstrate the ability to perform a detailed history and physical examination in a patient presenting with signs and symptoms of alcohol withdrawal, 2) investigate the broad differential diagnoses, including electrolyte abnormalities, trauma in the intoxicated patient, mild alcohol withdrawal, and delirium tremens, 3) list appropriate laboratory and imaging studies to include complete blood count (CBC), complete metabolic panel (CMP), magnesium level, computed tomography (CT) scan of the brain; 4) understand the management of hypoglycemia with concurrent administration of thiamine to prevent Wernicke’s encephalopathy and subsequent Korsakoff syndrome, 5) appropriately treat acute alcohol withdrawal with intravenous (IV) hydration and benzodiazepines, phenobarbital, or alternative medications, and 6) understanding the need for the complex management of these patients, appropriately disposition the patient to the intensive care unit after consulting with critical care specialists.

**Educational Methods:**

The case was written as an oral boards case to test learners in a simulated oral board format. In this manner, learners could be evaluated on their critical thinking skills one-on-one with an instructor, outside of the distractions of the emergency department. Oral board simulation can test multiple modalities, including data collection, data synthesization and pharmacologic treatment in order to assess residents’ overall clinical care and competence. Learners were assessed both by the instructor with immediate feedback, as well as by using Google forms to tie critical actions to Emergency Medicine Milestones. Results were compiled and used during clinical competency evaluations.

**Research Methods:**

Learners (n=40) and examiners were given the option to provide written feedback after the case was completed to assess for strengths and weaknesses of the oral boards case, and subsequent changes were made to improve the administration of the case.

**Results:**

Residents and medical students rated this highly and found this to be an enjoyable, yet still challenging, way to stay current on their management skills of alcohol withdrawal. Learners rated the session 4.6 out of 5 using a five-point Likert scale (5 being excellent) after the session was completed (n=25).

**Discussion:**

We found this oral board case to be an effective educational tool for reviewing alcohol use disorder among students and residents. Using an oral board case allows junior and senior residents to be tested quickly in a low-stakes environment. Learners and instructors both felt the content was appropriate, and using the completed forms in competency meetings improved the committee’s ability to assess residents on specific milestones. Though we initially wrote this case requiring the examinee to have advance knowledge of the Clinical Institute Withdrawal Assessment Alcohol Scale Revised (CIWA-Ar), this was not deemed essential to emergency medicine residents or faculty, and it was removed. The current case formatting represents a more realistic case presentation and critical actions.

**Topics:**

Alcohol withdrawal, electrolyte abnormalities, seizures, altered mental status.

## USER GUIDE

[Table t1-jetem-10-1-o1]

**Table t1-jetem-10-1-o1:** 

List of Resources:	
Abstract	1
User Guide	3
For Examiner Only	6
Oral Boards Assessment	12
Stimulus	15
Debriefing and Evaluation Pearls	29

**Learner Audience:**

Medical Students, Interns, Junior Residents, Senior Residents

**Time Required for Implementation:**

Case: 15 minutes

Debriefing: 10 minutes

Learners per instructor: 1, 1–2 others may observe

**Topics:**

Alcohol withdrawal, electrolyte abnormalities, seizures, altered mental status.

**Objectives:**

At the end of this oral boards session, learners will:

1. Demonstrate the ability to perform a detailed history and physical examination in a patient presenting with signs and symptoms of alcohol withdrawal.
2. Investigate the broad differential diagnoses, including electrolyte abnormalities, trauma in the intoxicated patient, mild alcohol withdrawal, and delirium tremens.
3. List appropriate laboratory and imaging studies to include complete blood count (CBC), complete metabolic panel (CMP), magnesium level, and computed tomography (CT) scan of the brain.
4. Understand the management of hypoglycemia with concurrent administration of thiamine to prevent Wernicke’s encephalopathy and subsequent Korsakoff syndrome.
5. Appropriately treat acute alcohol withdrawal with intravenous (IV) hydration and benzodiazepines, phenobarbital, or alternative medications.
6. Appropriately disposition the patient to the intensive care unit after consulting with critical care specialists, understanding the need for the complex management of these patients.

### Linked objectives, methods and results

Acute alcohol withdrawal is common in emergency medicine. Prompt diagnosis and early aggressive treatment reduces the risk of symptom progression to delirium tremens. The learner in this case should be able to synthesize the available historical and physical examination data in order to develop a broad differential diagnosis and identify signs and symptoms of acute alcohol withdrawal.

On initial evaluation, the learner will obtain a detailed history and complete physical examination which will suggest acute alcohol withdrawal (Objective 1). The learner should maintain a broad differential and obtain a thorough workup, including laboratory tests and imaging (Objectives 2 and 3). Recognize the need for point-of-care glucose testing in an altered patient and treat accordingly with glucose administration and concurrent administration of thiamine in the setting of alcohol abuse (Objective 4). Once the learners identify the patient is in acute alcohol withdrawal, they initiate treatment, to be guided by the clinical response to treatment (Objectives 3 and 5). The learner should provide appropriate disposition of the patient to an intensive care unit (Objective 6). Debriefing will ensure the learner can assimilate all of the clinical and lab findings in order to find the correct diagnosis.

### Recommended pre-reading for instructor

- Yancey J, Micciche D. Alcohol withdrawal syndrome: identification and management. emDocs Published May 2022. Accessed December 27, 2023. https://www.emdocs.net/alcohol-withdrawal-syndrome-identification-and-management/
- Newman RK, Stobart Gallagher MA, Gomez AE. Alcohol withdrawal. In: StatPearls. Treasure Island (FL): StatPearls Publishing; July 21, 2023.

### Results and tips for successful implementation

This model is best executed as an oral board single case examination. Learners should be directly observed by the instructor. Additional evaluators or instructors may also observe the case progression. This examination was tested during a mock oral board simulation, as well as during oral board practice sessions. Learners were evaluated using assessment forms that were created using Google forms. (https://docs.google.com/forms). The forms measured critical actions, which were also tied to Emergency Medicine Milestones (https://www.abem.org/public/docs/default-source/default-document-library/em-milestones.pdf?sfvrsn=e627c8f4_0). Using this format, oral board testing can be used to assess examinees’ clinical acumen and measure their progression along the emergency medicine milestones pathway.

This case was administered to 40 residents and used as one of three cases during an oral board simulation examination. Both interns and senior residents participated. Learner feedback for this case was positive, and learners were generally pleased with the case, rating it 4.6 out of 5 (n=25) on a 1–5 point Likert scale on overall enjoyment of the case (1 = poor, 5 = excellent). Learners overwhelmingly preferred the activity to a lecture format, rating 4.8 out of 5 (n=25) on a 1–5 Likert scale (1 = prefer lecture, 5 = prefer oral board testing). We also asked learners to provide open-ended comments, and these were generally positive, including, “I like being one-on-one with the attending to get a little more personal attention,” “This was my first time really managing alcohol withdrawal without a senior there, so it was helpful to go through the process,” and “I’d rather do this here than at ABEM general.”

The first iteration of this case was tested on five learners and required learners to calculate a CIWA-Ar score on their own. This requirement was removed after one session due to lack of utility; instructors and learners alike felt that this was both unnecessary from a learning standpoint and was unlikely to be tested in an oral board exam, and it was subsequently removed.

We feel this oral board case is highly testable and relevant in emergency medicine, and reinforces clinical and pharmacologic knowledge in a low-stakes setting.

### Pearls

- Alcohol withdrawal is a clinical diagnosis and should be taken into consideration within a broad infectious/toxic/metabolic differential so as not to prematurely close on an incorrect course of management.^3^
- Suspect alcohol withdrawal in a patient with a history of alcohol use disorder who reduces or ceases intake and presents with two or more of the following:
  ○ Increased hand tremor, autonomic hyperactivity, anxiety, agitation, nausea/vomiting, insomnia, hallucinations, generalized tonic-clonic seizures.
- Severity of alcohol withdrawal symptoms can be determined by the Clinical Institute Withdrawal Assessment of Alcohol Scale, revised (CIWA-Ar), with scores ranging from 0–67.^9^, ^10^
- Alcohol withdrawal symptoms typically begin a few hours after reduction or cessation in intake, peak at one to three days, and can last up to 14 days.^3^
- Life-threatening complications of alcohol withdrawal include seizures (6–15% of patients) and Delirium Tremens (3–5% of hospitalized patients).^2^
- Patients may exhibit malnourishment and have severe electrolyte imbalances. Life-threatening hypoglycemia, hypomagnesemia, hypokalemia and hyponatremia may need urgent correction. Consider co-administration of thiamine while administering glucose.^8^
- Symptom-triggered management, as directed by a patient’s CIWA-Ar score, is preferred over a fixed-dose strategy, and the intent is to achieve a lightly dozing but arousable state.^11^
- First-line treatment for alcohol withdrawal is the administration of benzodiazepines.
  ○ Diazepam^3^ –
    ▪ typical loading dose 10–20 mg IV
    ▪ quick onset, active metabolites, metabolized by liver and should be used cautiously in patients with decreased liver function.
  ○ Lorazepam^3^ –
    ▪ typical dose 2–4 mg IV
    ▪ quick onset, no active metabolites, metabolized by kidneys.
- Alternative treatment agents:
  ○ Phenobarbital^7^ –
    ▪ typical dose 10–15 mg/kg IV
    ▪ may also use bolus doses of 65 mg, 130 mg, and 260 mg IV
  ○ Propofol^3^ –
    ▪ 0.3–1.25 mg/kg/hr infusion typically used as adjunct for patients who require intubation and mechanical ventilation.
  ○ Ketamine^6^ infusion–
    ▪ usual dose 0.2 mg/kg/hr
  ○ second-line agent used in conjunction with other agents

## Figures and Tables

**Table t2-jetem-10-1-o1:** 

**Vitals:**	HR 107	BP 181/98	RR 14	Temp 36.4°C (97.5°F)	O_2_Sat 98% (room air)

**Table t3-jetem-10-1-o1:** 

	Milestone	Did not achieve level 1	Level 1	Level 2	Level 3
**1**	**Emergency Stabilization (PC1)**	□ **Did not achieve Level 1**	□ **Recognizes abnormal vital signs**	□ **Recognizes an unstable patient, requiring intervention** **Performs primary assessment** **Discerns data to formulate a diagnostic impression/plan**	□ **Manages and prioritizes critical actions in a critically ill patient** **Reassesses after implementing a stabilizing intervention**
**2**	**Performance of focused history and physical (PC2)**	□ **Did not achieve Level 1**	□ **Performs a reliable, comprehensive history and physical exam**	□ **Performs and communicates a focused history and physical exam based on chief complaint and urgent issues**	□ **Prioritizes essential components of history and physical exam given dynamic circumstances**
**3**	**Diagnostic studies (PC3)**	□ **Did not achieve Level 1**	□ **Determines the necessity of diagnostic studies**	□ **Orders appropriate diagnostic studies** **Performs appropriate bedside diagnostic studies/procedures**	□ **Prioritizes essential testing** **Interprets results of diagnostic studies** **Considers risks, benefits, contraindications, and alternatives to a diagnostic study or procedure**
**4**	**Diagnosis (PC4)**	□ **Did not achieve Level 1**	□ **Considers a list of potential diagnoses**	□ **Considers an appropriate list of potential diagnosis** **May or may not make correct diagnosis**	□ **Makes the appropriate diagnosis** **Considers other potential diagnoses, avoiding premature closure**
**5**	**Pharmacotherapy (PC5)**	□ **Did not achieve Level 1**	□ **Asks patient for drug allergies**	□ **Selects an appropriate medication for therapeutic intervention, considering potential adverse effects**	□ **Selects the most appropriate medication(s) and understands mechanism of action, effect, and potential side effects** **Considers and recognizes drug-drug interactions**
**6**	**Observation and reassessment (PC6)**	□ **Did not achieve Level 1**	□ **Reevaluates patient at least one time during the case**	□ **Reevaluates patient after most therapeutic interventions**	□ **Consistently evaluates the effectiveness of therapies at appropriate intervals**
**7**	**Disposition (PC7)**	□ **Did not achieve Level 1**	□ **Appropriately selects whether to admit or discharge the patient**	□ **Appropriately selects whether to admit or discharge** **Involves the expertise of some of the appropriate specialists**	□ **Educates the patient appropriately about their disposition** **Assigns patient to an appropriate level of care (ICU/Tele/Floor)** **Involves expertise of all appropriate specialists**
**22**	**Patient centered communication (ICS1)**	□ **Did not achieve level 1**	□ **Establishes rapport and demonstrates empathy to patient (and family)** **Listens effectively**	□ **Elicits patient’s reason for seeking health care**	□ **Manages patient expectations in a manner that minimizes potential for stress, conflict, and misunderstanding**.
**23**	**Team management (ICS2)**	□ **Did not achieve level 1**	□ **Recognizes other members of the patient care team during case (nurse, techs)**	□ **Communicates pertinent information to other healthcare colleagues**	□ **Communicates a clear, succinct, and appropriate handoff with specialists and other colleagues** **Communicates effectively with ancillary staff**

**Table t4-jetem-10-1-o1:** 

**#1**	**Patient Information Form**
**#2**	**Venous blood gas**
**#3**	**CBC**
**#4**	**BMP**
**#5**	**Urinalysis**
**#6**	**CXR**
**#7**	**Head-CT**
**#8**	**EKG**
**#9**	**Toxicology (serum and urine)**
**#10**	**Hepatic function panel**
**#11**	**Magnesium level**
**#12**	**Cardiac markers**
**#13**	**Coagulation panel**

**Table t5-jetem-10-1-o1:** 

**Patient’s Name:**	**Mark Jones**
**Age:**	**47**
**Gender:**	**M**
**Chief Complaint:**	**Intoxication**
**Person Providing History:**	**EMS, patient**
**Vital Signs:**	
**Temp:**	**36.4°C (97.5°F)**
**BP:**	**181/98**
**P:**	**107**
**RR:**	**14**
**Pulse Ox:**	**98% (room-air)**
**Weight:**	**93 kg (205 lb)**

**Table t6-jetem-10-1-o1:** 

pH	7.18
pCO2	38 mmHg
pO2	78 mmHg
HCO3	21 mmol/L
O2	sat 96%

**Table t7-jetem-10-1-o1:** 

WBC	14.4 × 10^3^/μL
Hgb	11.7 g/dL
Hct	34.2%
Platelets	290 × 10^3^/μL
Differential	
Neutrophils	58%
Lymphocytes	32%
Monocytes	4%
Eosinophils	3%
Bands	3%

**Table t8-jetem-10-1-o1:** 

Na	144 mEq/L
K	2.3 mEq/L
Cl	102 mEq/L
CO2	20 mEq/L
BUN	28 mg/dL
Cr	1.9 mg/dL
Glucose	133 mg/dL

**Table t9-jetem-10-1-o1:** 

Appearance	Clear
Color	Yellow
Glucose	Trace
Ketones	2+
Sp Gravity	1.020
Blood	Negative
pH	6.5
Protein	Negative
Nitrite	Negative
Leukocyte	Negative
WBC	0–2/hpf
RBC	0–2/hpf
Squamous Cells	15/hpf
Bacteria	0–2/hpf

**Figure f1-jetem-10-1-o1:**
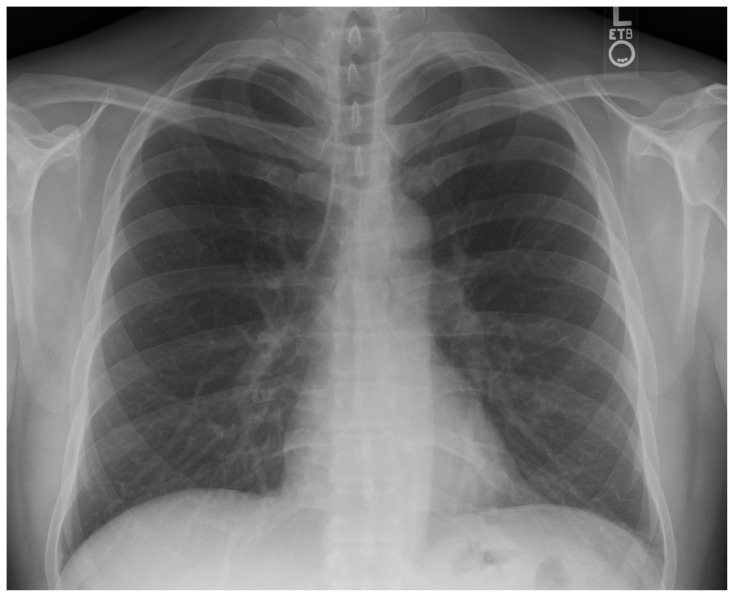
Image source: Stillwaterising. Normal PA chest radiograph. In: Wikimedia Common (https://commons.wikimedia.org/wiki/Main_Page) https://commons.wikimedia.org/wiki/File:Chest_Xray_PA_3-8-2010.png Published March, 2008. Accessed December 27, 2023. Public domain.

**Figure f2-jetem-10-1-o1:**
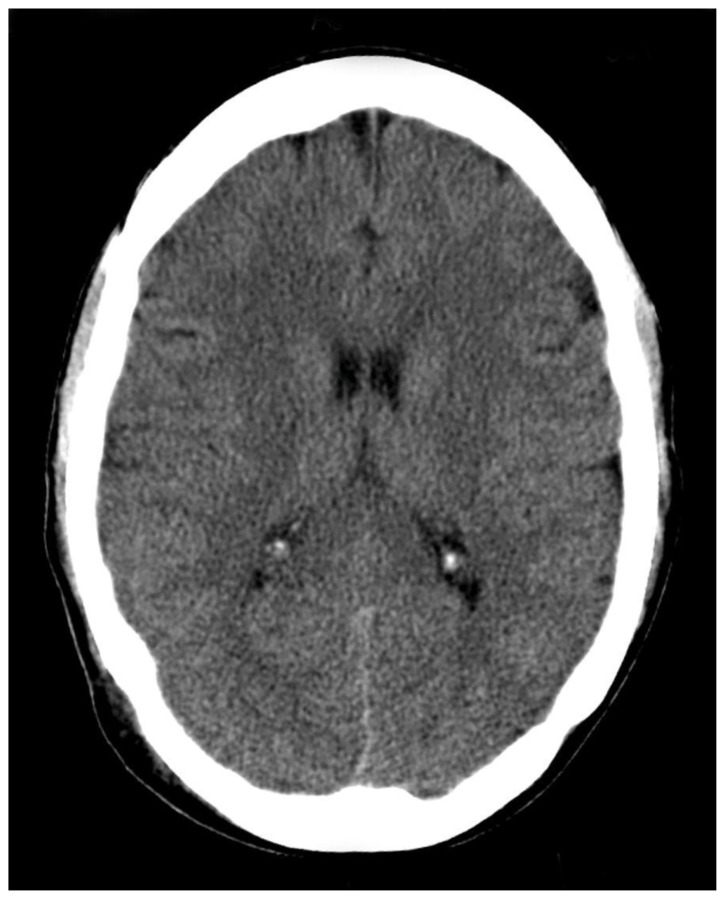
Image source: Filler G. Normal head-CT. In: Wikipedia.org. https://en.wikipedia.org/wiki/File:Brain_CT_scan.jpg. Published July 2009. Accessed December 27, 2023. CC-BY-SA 3.0.

**Figure f3-jetem-10-1-o1:**
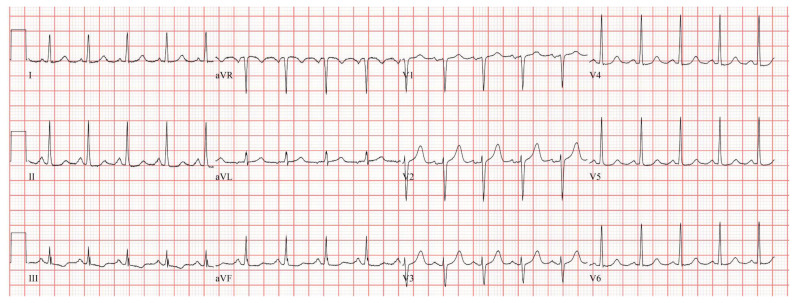
Image source: de Jong J. Sinus tachycardia. In: ECGpedia.org. https://en.ecgpedia.org/wiki/File:Sinustachycardia.jpg. Published November 2011. Accessed December 27, 2023. CC-BY-NC-SA 3.0

**Table t10-jetem-10-1-o1:** 

Amphetamines:	Negative
Barbiturates:	Negative
Benzodiazepines:	Negative
Cocaine:	Negative
Opiates:	Negative
Cannabinoids:	Positive

**Table t11-jetem-10-1-o1:** 

Acetaminophen:	<10 mCg/mL
Salicylates:	<15 mg/dL
Ethanol:	115 mg/dL

**Table t12-jetem-10-1-o1:** 

ALT	80 IU/L
AST	129 IU/L
AlkPhos	88 IU/L
T-bili	1.0 mg/dL
Direct Bilirubin	0.5 mg/dL

**Table t13-jetem-10-1-o1:** 

Magnesium	1.4 mEq/L

**Table t14-jetem-10-1-o1:** 

Troponin - I	24 ng/L
	Reference: < 20 ng/L – normal;
	20 ng/L – 80 ng/L – indeterminant;
	> 80 ng/L – elevated
BNP	71 pg/ml
	Reference: < 90 pg/ml – normal

**Table t15-jetem-10-1-o1:** 

PTT:	29.1 seconds
PT:	15.1 seconds
INR:	1.1

## FOR EXAMINER ONLY

### Oral Case Summary

**Diagnosis:** Alcohol withdrawal with delirium tremens

**Case Summary:** This is a case of a 47-year-old male patient who presents to the ED via EMS for a seizure episode. The patient was found at a local gas station and the owner called 911 after witnessing the patient fall to the ground and have shaking movements of his arms and legs and confusion after the episode. EMS states that the patient is well-known to them as someone who is frequently intoxicated. The patient is able to state his name but appears to be responding to internal stimuli and is having visual hallucinations with episodes of vomiting. He is suffering from acute alcohol withdrawal and will have additional seizures in the ED if not treated with a benzodiazepine, barbiturate, or ketamine. If not treated urgently, the patient will require intubation and mechanical ventilation. Additionally, electrolytes should be corrected, and the patient will require ICU admission.

**Order of Case:** The patient will present to the ED via EMS and initially the EMS crew is “not excited” about the patient. They will discuss that he is a “frequent flier” and that he is usually intoxicated, and they think he is drunk again tonight. The patient is unable to provide accurate history and appears to be responding to internal stimuli. He complains of headache and vomits during the examination. The learner should request a CIWA-Ar score to be calculated to help guide therapy. The RN will provide a CIWA score of 15 when prompted.

Alcohol withdrawal syndrome should be recognized, and the patient should be treated with IV benzodiazepines, phenobarbital, or ketamine initially. If left untreated, the patient will seize in the ED until one of these medications is provided. If the syndrome continues to not be recognized, the patient will again seize until general anesthesia, intubation, and mechanical ventilation is necessary.

Testing in the ED should focus on both the trauma of the fall and the evaluation of electrolyte abnormalities. The patient will be found to have severe hypokalemia and hypomagnesemia, and these electrolytes will need to be replaced intravenously. The patient should be dispositioned to the ICU after the resuscitation is completed.

**Disposition:** Admit to intensive care unit

**Critical Actions:**

1. Administer 1 ampule (25 grams) of dextrose (D50) with associated IV thiamine (500mg, may request dose from pharmacy)
2. Establish severe alcohol withdrawal and delirium tremens as cause of signs and symptoms, and administer a benzodiazepine, phenobarbital, or ketamine for stabilization.
3. Obtain CT-head to assess for trauma.
4. Discover electrolyte derangement and replace magnesium and potassium intravenously.
5. Admit to intensive care unit.

**Dangerous Actions:**

1. Sending the patient to CT-scan without first initiating treatment for alcohol withdrawal.
2. Sending the patient to a medical floor instead of the intensive care unit.

### Historical Information

**Chief Complaint:** “He is drunk.”

**History of present illness:** A 47-year-old male patient is brought to the ED via EMS. Report is that he was found to be confused and intoxicated at a local gas station. He fell to the ground and had a seizure, and a bystander called 911. The patient is oriented to himself only and is not able to provide accurate history. He is complaining of a headache and has an episode of vomiting while being questioned. If asked to remain to answer questions, EMS will state that the patient is well-known to them, and they are frequently called to transport him to local hospitals while intoxicated. He was apparently stumbling around the gas station earlier and appeared intoxicated prior to the incident. If requested, EMS reports a fingerstick blood glucose level of 57 mg/dL.

**Past Medical history:** Alcohol use disorder, hypertension

**Past Surgical history:** Unknown

**Patient’s Medications:** Not taking any

**Allergies:** No known drug allergies

**Social history:**

- Alcohol: Regularly
- Tobacco: Regularly
- Drug use: Marijuana

**Family history:** None relevant

### Physical Exam Information

[Table t2-jetem-10-1-o1]

**Weight:** 93 kg (205 lb)

**General appearance:** Unkempt, chronically ill appearing, disheveled

**Primary survey:**

- **Airway:** Intact, no stridor, protecting airway
- **Breathing:** Diminished bilaterally, decreased inspiratory effort, no wheezes
- **Circulation:** Intact peripheral pulses, tachycardic, normal heart tones

**Physical examination:**

- **General appearance:** Unkempt, chronically ill appearing, disheveled, somnolent but rousable to voice
- **HEENT:**
  ○ **Head:** Abrasion to the left side of the forehead, no lacerations, no active bleeding
  ○ **Eyes:** Pupils equal, 3–4 mm bilaterally, reactive to light, no nystagmus
  ○ **Ears:** Within normal limits
  ○ **Nose:** Within normal limits
  ○ **Oropharynx/Throat:** Mucous membranes dry, mild tongue fasciculations
- **Neck:** Within normal limits
- **Chest:** Diminished breath sounds bilaterally, no wheezes, non-labored breathing, no chest wall tenderness or crepitus
- **Cardiovascular:** Tachycardic, heart tones unremarkable, 2+ Dorsalis Pedis (DP) and Posterior Tibial (PT) Pulses bilaterally
- **Abdominal/GI:** Soft, non-tender, non-distended
- **Genitourinary:** Within normal limits
- **Rectal:** If asked, within normal limits with negative guaiac
- **Extremities:** Moving extremities spontaneously, no deformities or overt signs of trauma, appears to have equal strength in all extremities, tremulous with arms extended
- **Back:** Within normal limits
- **Neuro:** Somnolent, but rousable to verbal and/or painful stimuli, pupils equal and reactive bilaterally, no slurred speech, no facial asymmetry, uvula and tongue are midline, moves all extremities to command, oriented to self only, and not place and time, no obvious focal neurologic deficits noted
  ○ If requested, GCS=13 (E=3, V=4, M=6)
- **Skin:** Scattered bruises and hematomas on the extremities of various stages, no open wounds, no rashes, palms damp
- **Lymph:** Within normal limits
- **Psych:** Disoriented to time and place, appears confused. Is concerned about a feeling of something crawling on his skin. Is anxious.

### Critical Actions and Cueing Guidelines

1. **Critical Action 1**
  *Administer 1 ampule (25 grams) of dextrose (D50) concurrently with IV thiamine.*
  - Cueing Guideline (if applicable):
    *RN will ask, “Did EMS obtain a fingerstick before the patient arrived?”*
  - *RN may state, “Usually we also give a vitamin, but I can’t remember which one.”*
2. **Critical Action 2**
  *Establish severe alcohol withdrawal and delirium tremens as cause of signs and symptoms, and administer a benzodiazepine, phenobarbital or ketamine for stabilization.*
  - Cueing Guideline (if applicable):
    *RN may ask, “Can we give him anything to prevent any more seizures?”*
3. **Critical Action 3**
  *Obtain CT-head to assess for trauma.*
  - Cueing Guideline (if applicable):
    *RN may state that the patient didn’t have abrasions to forehead when he/she saw the patient intoxicated yesterday.*
4. **Critical Action 4**
  *Discover electrolyte derangement and replace magnesium and potassium intravenously.*
  - Cueing Guideline (if applicable): N/A
5. **Critical Action 5**
  *Admit to intensive care unit.*
  - Cueing Guideline (if applicable):
    *If learner calls for a “floor bed” (or equivalent), the admitting team may ask if the patient is hemodynamically normal enough for the floor, or if he is on any drips, eg, potassium or magnesium.*

## ORAL BOARDS ASSESSMENT

*Alcohol Withdrawal*

Learner: _________________________________________

**Critical Actions:**

□ Administer 1 ampule (25 grams) of dextrose (D50) with associated IV thiamine *(500mg, may request dose from pharmacy).*
□ Establish severe alcohol withdrawal and delirium tremens as cause of signs and symptoms, and administer a benzodiazepine, phenobarbital, or ketamine for stabilization.
□ Obtain CT-head to assess for trauma.
□ Discover electrolyte derangement and replace magnesium and potassium intravenously.
□ Admit to intensive care unit.

**Summative and formative comments:**

**Milestone assessment:** [Table t3-jetem-10-1-o1]

### Stimulus Inventory

[Table t4-jetem-10-1-o1]

#### Stimulus #1

##### Patient Information

[Table t5-jetem-10-1-o1]

#### Stimulus #2

##### Venous Blood Gas

[Table t6-jetem-10-1-o1]

#### Stimulus #3

##### CBC

[Table t7-jetem-10-1-o1]

#### Stimulus #4

##### BMP

[Table t8-jetem-10-1-o1]

#### Stimulus #5

##### Urinalysis

[Table t9-jetem-10-1-o1]

#### Stimulus #6

##### CXR

[Fig f1-jetem-10-1-o1]

#### Stimulus #7

##### Non-contrast CT-head

[Fig f2-jetem-10-1-o1]

#### Stimulus #8

##### EKG

[Fig f3-jetem-10-1-o1]

#### Stimulus #9

##### Toxicology (Urine and Serum)

**Urine Drug Screen:** [Table t10-jetem-10-1-o1]

**Serum Toxicology:** [Table t11-jetem-10-1-o1]

#### Stimulus #10

##### Hepatic function panel

[Table t12-jetem-10-1-o1]

#### Stimulus #11

##### Magnesium Level

[Table t13-jetem-10-1-o1]

#### Stimulus #12

##### Cardiac Markers

[Table t14-jetem-10-1-o1]

#### Stimulus #13

##### Coagulation panel

[Table t15-jetem-10-1-o1]

## DEBRIEFING AND EVALUATION PEARLS

### Alcohol Withdrawal

- Alcohol withdrawal is a clinical diagnosis and should be taken into consideration within a broad infectious/toxic/metabolic differential so as not to prematurely close on an incorrect course of management.^3^
- Suspect alcohol withdrawal in a patient with a history of alcohol use disorder who reduces or ceases intake and presents with two or more of the following:
  ○ Increased hand tremor, autonomic hyperactivity, anxiety, agitation, nausea/vomiting, insomnia, hallucinations, generalized tonic-clonic seizures.
- Severity of alcohol withdrawal symptoms can be determined by the Clinical Institute Withdrawal Assessment of Alcohol Scale, revised (CIWA-Ar), with scores ranging from 0–67.^9^, ^10^
- Alcohol withdrawal symptoms typically begin a few hours after reduction or cessation in intake, peak at one to three days, and can last up to 14 days.^3^
- Life-threatening complications of alcohol withdrawal include seizures (6–15% of patients) and Delirium Tremens (3–5% of hospitalized patients).^2^
- Patients may exhibit malnourishment and have severe electrolyte imbalances. Life-threatening hypoglycemia, hypomagnesemia, hypokalemia and hyponatremia may need urgent correction. Consider co-administration of thiamine while administering glucose.^8^
- Symptom-triggered management, as directed by a patient’s CIWA-Ar score, is preferred over a fixed-dose strategy, and the intent is to achieve a lightly dozing but arousable state.^11^
- First-line treatment for alcohol withdrawal is the administration of benzodiazepines.
  ○ Diazepam^3^ –
    ▪ typical loading dose 10–20 mg IV
    ▪ quick onset, active metabolites, metabolized by liver and should be used cautiously in patients with decreased liver function.
  ○ Lorazepam^3^ –
    ▪ typical dose 2–4 mg IV
    ▪ quick onset, no active metabolites, metabolized by kidneys.
- Alternative treatment agents:
  ○ Phenobarbital^7^ –
    ▪ typical dose 10–15 mg/kg IV
    ▪ may also use bolus doses of 65 mg, 130 mg, and 260 mg IV
  ○ Propofol^3^ –
    ▪ 0.3–1.25 mg/kg/hr infusion typically used as adjunct for patients who require intubation and mechanical ventilation.
  ○ Ketamine^6^ infusion–
    ▪ usual dose 0.2 mg/kg/hr
  ○ second-line agent used in conjunction with other agents
